# White-centered retinal hemorrhage

**DOI:** 10.11604/pamj.2014.17.233.4038

**Published:** 2014-03-27

**Authors:** Soufiane Berradi, Rajae Daoudi

**Affiliations:** 1 Université Mohammed V Souissi, Service d’‘Ophtalmologie A de l’‘Hôpital des Spécialités, Centre Hospitalier Universitaire, Rabat, Maroc

**Keywords:** Retinal hemorrhage, visual acuity, acute lymphoblastic leukemia

## Image in medicine

A 37-year-old woman presented with unilateral sudden drop in visual acuity of the right eye up to a week. Anamnesis revealed episodes of fever and headache. Examination showed visual acuity of 20/400 in the right eye and 20/20 in the left eye. The fundus examination showed white-centered retinal hemorrhage (Panel A, black arrow) in his right eye. The white center is due to cellular debris, to capillary emboli or leukemic infiltrates. Angiography showed a mask effect on autofluorescence sequence (Panel B, white arrow). The visual field showed a right central scotoma. The etiological investigation revealed a leukocytosis, anemia and thrombocytopenia, in favor of acute lymphoblastic leukemia which is a malignant disease of hematopoietic tissue characterized by clonal proliferation of hematopoietic precursors derived from the lymphoid lineage. The patient was referred to the hematology department for chemotherapy. Two weeks later, visual acuity recovered to 20/50 in his right eye and hemorrhage significantly regressed.

**Figure 1:**
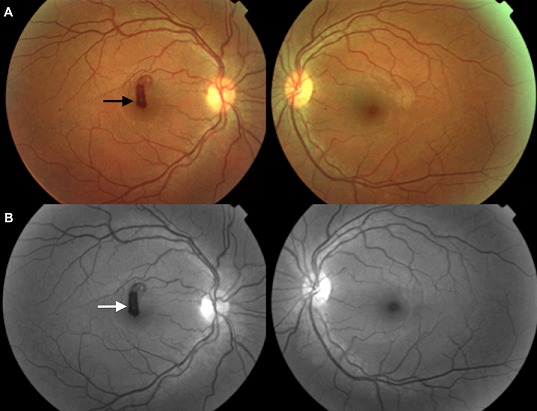
Panel A, black arrow: Fundus examination showing white-centered retinal hemorrhage in the right eye; Panel B, white arrow: Angiography showing a mask effect on autofluorescence sequence

